# American Medical Association

**Published:** 1888-06

**Authors:** 


### SOCIETY REPORTS.

### AMERICAN MEDICAL ASSOCIATION.

GENERAL SESSION.

FIRST DAY—May 8.

Thirty-ninth annual session was held in Cincinnati, Ohio, May 8, 9, 10, and 11.

The Association was called to order by Professor W. W. Dawson, Chairman of the Committee of Arrangements.

Addresses of welcome were delivered by Mayor Amor Smith, and Professor C. G. Comegys, the former recalling the meetings of the Association in Cincinnati, in 1850 and 1867.

At the close of these addresses some miscellaneous business was transacted, and then

Dr. A. Y. P. Garnett, of Washington, D. C., the President of the Association, delivered an address upon The Mission of the American Medical Association. The address reviewed the history of medical education in the United States, and embodied some propositions intended to advance the standard of medical education, and which would practically place the whole matter in the hands of the American Medical Association. The following is a synopsis of the address:

Our annual meetings must not be regarded solely for the purpose of considering the science of medicine either in its theoretical or practical aspect and supplemented by the pleasures of social intercourse. While we have ample cause to be gratified with the rapid progress made by some departments of medicine in this country within the last decade under the direction and stimulus of this Association, we are still confronted by the fact that very little has been effected in the direction of a general reform in the medical schools.

We all know that this subject of medical education and medical license has been an almost endless theme for discussion, coeval, indeed, with the establishment of medical schools in America, and more particularly has it been earnestly and ably considered since the organization of this Association; yet the question still presents itself, What can the American Medical Association do to further raise and promote a higher standard of medical education in the United States, to curtail the number of medical schools?

Permit me to disclaim here any purpose or desire to impugn the motives or loyalty to our profession of these distinguished representatives of our medical colleges. From my standpoint alone do I offer this hypothetical criticism upon their conduct in this regard; and while I cheerfully concede to them the honor and merit of having largely participated in laying the foundation of this Association, and having subsequently sustained its character, influence, and power as an agent for the general welfare of the medical profession in the United States, I can not permit myself to ignore the fact that they have not, collectively and unitedly, co-operated and wielded the power which they indisputably possess to advance and elevate our standard of medical education.

While it can not be denied that there are medical colleges in this country unsurpassed by those of Europe in their essential appointments and facilities for the education of medical students, the equally undeniable facts remain that we fall far behind them in the systems of study pursued.

We have waited, and nearly half a century has rolled by in anxious expectation of seeing these educational centres take the initiative in pushing forward this great object, an object which in reality dominated all other considerations at the very birth of this Association. Have those expectations been realized? On the contrary, with a few notable exceptions, they have turned a deaf ear to the repeated suggestions and earnest demands of this body.

That the professions of law and medicine are over-crowded in this country, no man of common observation will deny. The ratio of professional men in the United States to the population exceeds that of any other country in the civilized world, so that any legitimate means of checking this evil which can be devised and carried into practical effect must be hailed by the medical world, as well as the general public, as an inestimable boon.

Inspired by the hope of initiating some practical move in the direction of educational reform, I respectfully submit for your consideration the following suggestions:

*Proposition* 1. That a standing committee, to be called a committee on legislation, be appointed for each State, Territory, and the District of Columbia, to consist of five members of the medical profession in good standing, three of whom shall have no official connection with any medical school or college, whose duty it shall be to carry out as far as possible the following instructions:

*First.* That each one of said committees, or a majority thereof, shall attend the sessions of their respective Legislatures, or from time to time as their duties may require, for the purpose of using all honorable means looking to the reduction of the number of medical schools in the United States, and a consequent diminution in the annual number of medical graduates; that as a practical measure to this end they urge the passage of a law requiring that in the future granting of charters for creating medical schools there shall be a clause in every such charter requiring that all schools or colleges thus created shall demand a full term of four years' study before granting a diploma to any student thereof, and that no student shall be admitted to matriculate who has not passed a satisfactory examination, both oral and written, in the ordinary branches of academic study; and further, that any college failing to show a greater number than fifty matriculates annually for three consecutive years shall forfeit its charter and be abolished.

*Second.* That they use all diligent effort to secure an ordinance creating in each State and Territory where no such board at present exists, and the District of Columbia, a board of medical examiners, which shall have no connection with any medical school, and which shall be required to examine all applicants for license to practice medicine in their respective States, Territories, and the District of Columbia; and that any person who may be detected in practicing any branch of the healing art without a license granted by said board, shall be subject to such penalties as the law may provide. That this committee may be authorized by statute to select and nominate to the Governors of the States, Territories, and the District of Columbia seven competent and learned members of the medical profession to constitute said Board of Examiners, who shall have the exclusive power to issue licenses to practice the art and science of medicine and surgery.

*Third.* That the chairman of said committees of five be required to submit at each annual meeting of this Association a report embracing a full statement of what has been accomplished by each.

*Proposition* 2. That the faculties of the several medical schools within the limits of the United States be once more urgently requested to call a convention at some central point, for the purpose of consultation and adopting some general and uniform system of medical education more comprehensive and rigid in its requirements, and more in accord with the spirit of the age and the advanced progress of medical science, suggesting a four years' term of study, the requirement of a preliminary education, including some knowledge of the classics.

That any college or school which shall refuse to enter into such an arrangement as may be decided upon by said convention shall be excluded from all connection with the American Medical Association, and its alumni not recognized as members of the regular profession.

I am aware that these suggestions embrace some very radical and seemingly impracticable changes; but as they point, in my judgment, to the right direction, I trust that they may not prove to be seeds sown upon barren soil. Be that as it may, I shall at least enjoy the consciousness of having honestly, conscientiously, and fearlessly met the great and pressing issue of the day within the domain of our profession, and of having executed, to the best of my ability, the most important duty imposed by the responsible position to which you have elevated me.

### GENERAL SESSION.

#### SECOND DAY—May 9.

On the opening of the session the secretary announced the names of the *Nominating Committee* which had been selected.

The *Address in Medicine* was then delivered by Professor Roberts Bartholow, of Philadelphia, Pennsylvania, in which he alluded to the flippant manner in which the secular press too often alludes to the medical profession and its work. In illustration of this he spoke of the manner in which the daily press spoke of the work of the last International Medical Congress held in Washington last year, which, he said, "in some respects was the most important of the series."

He then assigned some of the reasons to which he attributes that habit. He said:

The causes of this state of things are not far to seek. Every one here present must be familiar with them. I purpose only to consider those which have to do with the therapeutic art—the most important.

Not a little of the ignorance, the crudities, the vague theories of the eighteenth century still linger in the nineteenth century near its close. It is yet understood by many that the therapeutic art is based on some *ism* or dogma. "Allopath" and "homœopath" are still bandied about as having vital power, when they were long since relegated to the lumber-room of the past by modern science. We yet hear of "new school" and "old school," as if notions brought out near the close of the last century, compounded of the vagaries of Hahnemann and the mysticism of Mesmer, could still continue guides to practice. The contentions of so-called "schools," the noisy demonstrations of the Thompsonians, and eclectics, and physio-medicals; the rabid water-cure, and the innumerable special cures, have produced in the popular mind a distrust of all systems.

The cry of "new school" has proved the most sympathetic; but the attractive sugar pellets, and tasteless liquids, carry with them their own destruction, for, in the deeper consciousness of men, lies the fatal objection that great results are not to be accomplished by such slender means. Hence, left to its own course, homœopathy has practically died out on the continent. On this side of the ocean it still maintains a certain influence, because of social conditions and prejudices that are only possible in free communities.

The success of such wretched puerilities, such inanities as the homœopathic practice consists of, does more to lower the position of the medical profession than any other cause. The one remedy for existing ills, and to put the medical profession in its proper attitude as a scientific body, is to improve the art.

As a teacher of therapeutics for many years, I could not fail to observe that the attainments of the profession in respect to the powers of medicines are not as fully developed as they ought to be. They do not have that familiarity with the physiological powers of drugs which the effective use of remedies demands. It thus happens that the highest precision is not attained. Vague notions take the place of scientific accuracy, when such has been the progress in this department that a considerable degree of certainty should be the rule.

It must be admitted that the acquisition of therapeutical skill has been greatly hampered by the complexity of the materials. The barnacles of a century have been accumulating on the framework, and the new knowledge has been thrust into the crevices of old notions until all is made to appear confused and uncertain.

The subject should be divested of its superfluities. The list of preparations given in the *U. S. Pharmacopœia*—our only official authority—can be greatly curtailed with advantage. Remedies long in use and of comparatively little value are overloaded with formulæ. The preparations of iron given by the *Pharmacopœia* are thirty-eight in number, of mercury twenty-five, of rhubarb fifteen, of aloes as many. One-third of these could perform the duty of all. The botanical and pharmaceutical details are such that to master them would require the whole of the time given to the medical studies. If obtained, such knowledge is of small value to physicians; therefore it should be turned over to the pharmacist, to whom it properly belongs.

After the delivery of the address the secretary of the *Board of Trustees of the Journal Association* read the annual report of the board, in which it was stated that the *Journal* had been promptly issued throughout the year; that it remains under the same editorial management, and that it has its own office of publication in Chicago, Illinois. A report of the receipts and of the expenditures was made, showing that the *Journal* is more than self-sustaining.

The chairman of the *Committee on Dietetics* made the report of the committee, and, on motion, it was ordered that the committee be continued, and that it have power to increase its own membership.

The amendments to the constitution of the Association offered at the last annual meeting were then called up and read. The amendment allowing physicians to become *members by application* was unanimously adopted.

The amendment regarding the *Board of Trustees*, to consist of nine members, was also unanimously adopted.

The third amendment, relative to the superseding of the *Nominating Committee* was read, and, after discussion, it was agreed to print the amendments and to make it the special order for Thursday, at 12 m.

GENERAL SESSION.

THIRD DAY—May 10.

On the assembling of the Association the secretary read the resolutions adopted by the *State Medical Society of Arkansas* relative to *the patronage given to charlatanism by the religious press of our country.*

By vote the Association indorsed the condemnation of the practice as expressed in the resolutions read.

The *report of the Nominating Committee* was then made.

OFFICERS FOR 1889.

President—W. W. Dawson, Cincinnati, Ohio.

First Vice-President—W. L. Schneck, Kansas City, Missouri.

Second Vice-President—F. Woodbury, Philadelphia, Pennsylvania.

Third Vice-President—H. O. Walker, Detroit, Michigan.

Fourth Vice-President—J. W. Bailey, Georgia.

Treasurer—R. J. Dunglison, Philadelphia, Pennsylvania.

Permanent Secretary—W. B. Atkinson, Philadelphia, Pennsylvania.

Librarian—C. H. A. Kleinschmidt, Washington, D. C.

Next place of meeting, Newport, Rhode Island; time of meeting, first Tuesday in June, 1889.

Chairman of *The Committee of Arrangements*—H. R. Storer, Newport.

To deliver the *Address in Medicine*, Professor William Pepper, Philadelphia, Pennsylvania.

The *Address in Surgery*, Professor P. S. Connor, Cincinnati, Ohio.

The *Address in State Medicine*, Professor W. H. Welch, Baltimore, Maryland.

The nominations made in the report were confirmed by vote.

The *Address in Surgery* was then delivered by Dr. E. M. Moore, of Rochester, New York.

After giving, in general outline, a review of early and modern surgery, and noting prominent points of the progress of surgery in the *United States* during the last half century, he spoke of the influence of the discoveries of Pasteur and Koch, which constitutes the most interesting and valuable part of the address.

The microbal discoveries of Pasteur and Koch and their disciples have placed all our therapeutics on a new basis. No one knows when a real discovery is made how far it will reach. It seems to run like a thread through the woof of all knowledge. How can we measure the scope of a piece of glass ground to a lens? The spectroscope was a mere philosophic toy at first. What now? The analyst of the sun as well as of the blood. No one can measure the possible triumphs of surgery. That which was excellent yesterday must be abandoned to-day.

Who has not dreaded the care of a compound fracture of the thigh? But now we have a report by Professor Mac Ewan of a thousand cases of osteotomy and no bad results. Who a few years since could have accepted the statement that the bone could be cut and crushed, the surface broken and driven into the texture of the bone above and below and yet remain absolutely free from danger of necrosis and ready for union? The operation is essentially subcutaneous. But Dr. Hahn, of Berlin, boldly incises the soft parts and exposes the surface of the tibia under a stream of mercuric solution, regarding this operation as secure as the subcutaneous cut, and as the parts can be seen they are more susceptible of proper management. In all these cases merely the quiet necessary to physiological repair with its antiseptic covering comprises the after treatment. Its simplicity and rapidity of result amazes one.

The report of the *Committee on the Rush Monument* was then read, showing the work done by the committee, and the amount of money received for the fund.

The report of the treasurer and of the librarian were next made.

The hour having arrived for he special order of business, the consideration of the third amendment to the constitution, it was moved and carried that further consideration of the subject be postponed for one year.

Another amendment was offered, to be considered at the next annual meeting—to "Strike out the last clause or paragraph of Section VII," relating to individually affixing names to the constitution and regulations of the Association.

Another amendment was offered for consideration at the next annual meeting, as follows:

There shall be created a Section of Pharmacy and Materia Medica, which shall have its own autonomy in like manner as the Section in Dental and Oral Surgery.

Reputable members of State pharmaceutical associations shall be eligible as members of the same on presentation of credentials from their State secretary, but shall have no voice in the general sessions of the Association. The Section of Surgery shall hereafter be denominated the Section of Surgery and Gynæcology. There shall be created a Section of Anatomy and Physiology. The Section of Obstetrics and Diseases of Women shall be abolished. The Section of Diseases of Children shall hereafter be denominated the Section of Obstetrics and Pædiatrics. The Section of Dermatology and Syphilography shall hereafter be denominated the Section of Dermatology and Genito-Urinary Diseases. The Section of Medical Jurisprudence shall hereafter be denominated the Section of Mental and Nervous Diseases. The Section of State Medicine shall hereafter be denominated the Section of State Medicine and Medical Jurisprudence. The Section of Practice of Medicine, Materia Medica, and Physiology shall hereafter be denominated the Section of Internal Medicine.

On motion, it was

*Resolved*, That in future, each delegate or permanent member, shall, when he registers, also record the name of the section, if any, that he will attend, and in which he will cast his vote for section officers.

It was stated that it was offered because it was found that members voted in several sections, thus aiding in the election of officers of more than one section.

It was moved to rescind the action permitting the sections to meet at 11 a. m.

After some discussion, it was adopted.

GENERAL SESSION.

FOURTH DAY—May 11.

On the assembling of the Association the *Address in State Medicine* was delivered by Dr. H. P. Walcott, of Cambridge, Massachusetts, who gave an interesting account of the work of the State Board of Health of Massachusetts, now in the twentieth year of its existence, in which he stated that the board has "a fairly kept record of our vital statistics from the year 1842 to the present time." From the experience of the board in its twenty years of existence, useful lessons in preventive medicine had been learned, and he essayed to picture some of them, embracing the protection of water supplies, and better drainage for large cities.

The difficulties attending legislation for proper sanitary regulations were considered, including questions of quarantine.

Many of our law-makers have discovered, I believe, no other authority for sanitary legislation on the part of the General Government than that which is contained in the section of the Constitution which declares that Congress, among other functions, shall have power "to regulate commerce." There does not appear to be any clearly expressed provision in the above-named document for the appropriation of money to help exterminate contagious pluro-pneumonia in cattle, and yet the Forty-ninth Congress did appropriate $500,000 for that purpose, and wisely, I am sure we shall all say. I read, however, in this same section of the Constitution that Congress shall also have power to provide "for the general welfare of the United States."

In our own day we have seen National, State, and local laws swept away by cholera and yellow fever. Let us hope then that some ingenious law-maker may find in these words a sufficient authority for the expenditure of enough of the public money, at least, to secure some of those thorough investigations of preventable disease which have characterized the work of the Gesundheitsamt in Germany and the Local Government Board in England.

When it can be deliberately stated—as I believe the experience of the last ten years makes it possible for us to state—that improved methods of research, that the knowledge already gained, have made it almost certain that co-ordinated investigations properly encouraged and supported by such aid as only a great government can give, would lead to the discovery of the causes of yellow fever and malarial fever in the first place, and in the second, to rational methods of dealing with these most formidable scourges of the land in the way of prevention or of cure ; then, I say, it does seem in the highest degree absurd that any form of government should deliberately deprive itself of the sanitary gains which contribute so directly to the preservation of "life, liberty, and the pursuit of happiness."

It has been well said by one of the most eminent of the executive officers of health, Dr. Russell, of Glasgow, that "nothing is more conspicuous than the helplessness of the individual under the conditions of civilized life to secure the physical basis of health."

How can any single individual in a crowded city make any successful effort to improve, or even to ascertain, the quality of the public water supply upon which he is exclusively dependent? What can he do to secure a sufficiently prompt and safe removal of the waste materials of his daily life? How can he analyze the ingeniously sophisticated articles of food or medicine which may represent the ingenious employment of all the scientific skill that selfish capital can purchase? How vain it would be for him to attempt to prevent the crowding of the city, or the contamination of the air which he is obliged to breathe. Lastly, how can he recognize the presence of communicable disease, and recognizing, keep it at a distance?

There is no help but in co-operation on the most extended scale possible, individual, municipal, State, and National. The individual must be compelled to give up his liberty to injure his neighbor. The great city must be restrained from converting the stream that flows past it into a sewer that shall poison the small villages that cluster about its banks lower down in its course. The country farmhouse must no longer, when a case of enteric fever occurs there, be a menace to the water that supplies a thousand city houses. No State should permit its own causes of disease, whatever they are, persons or things, to be transported into another State, nor pollute the common water supply. Lastly, the General Government should take cognizance of those causes of disease which can be controlled by no other power, or which are so general that the responsibility for them is National, or so overwhelming in their ravages that smaller political bodies have lost the power, if not the will, to protect the people.

Voluntary association on the part of persons, towns, States, or Nations even, will never establish a sufficient safeguard when one dishonest or ignorant associate has it in his power to nullify all the effects of a neighbor's spirit of self-sacrifice and restraint.

Epidemic disease is an evil that can not be left to work out its own cure; it widens its circle too rapidly, and the time is long past when any quarantine, using the word in its older sense, would be sufficient to restrain a pestilence within the limits of the district whose sanitary crimes might have fairly deserved some punishment.

How, then, shall we organize for the protection of the public health?

For the individual, that he may not injure his neighbor through his own ignorance or that of his adviser, let the State give him some assurance that the legally used title of physician designates a person sufficiently qualified to give advice for the prevention and cure of disease—and a certainty, also, that the quack who steals the title shall be punished.

Establish, by direct provision of State law, local health authorities for each well-defined unit in our various forms of local government—village, town, city, or county. Place in the hands of these authorities the execution of the laws of the State and all local ordinances and regulations pertaining to the public health, impress upon them the duties of watching all those conditions which immediately affect the health of the people: water supply, sewerage, sanitary condition of school-houses and scholars, factories, tenement houses and the markets, periodic inspection of the inhabitants to prevent the neglect of vaccination, house to house inspection at least annually. In brief, they should have all the powers which society assumes for protecting the public health.

To meet the possibility that some districts may not have intelligence enough to appreciate, or that powerful commercial or other interests may interfere with, these laws, there should be a State authority with ample powers.

This would be called upon for the purpose of harmonizing the action of the various local boards, where the interests of various localities were involved. It should have co ordinate power with all local boards in case of communicable diseases, in order that its action may be prompt and salutary. It should be enabled to carry on scientific investigations as to the causes of disease, to suppress noxious and offensive trades, as it will often happen that some great industry will possess in a community an influence sufficient to control the local board of health. This condition did in fact exist in Massachussetts, and no action of our State Board of Health in its earlier years was more heartily commended than that which transformed a town, pestilent and disgusting from some fifty slaughter-houses, into an attractive suburb of Boston. The business of slaughtering and rendering is now, in consequence of the action of the board, carried on in an abattoir entirely without offense.

This authority should also have the supervision of the laws for the prevention of the adulteration of food and drugs.

It should have the general oversight of the public water supplies.

It should have charge of the registration of vital statistics, and of the investigations of the diseases of animals so far as they affect the health of man.

The educational element should be always present in the work of the State and local boards of health; and, as in all other affairs of life, the value of the lesson will stand generally in direct relation to the cost of it. The person who receives the benefit of a sanitary improvement should pay, if able to do so, a part, at least, of the cost, and should never be permitted to lose a sense of the obligation of preventing the spread to his neighbor of unsanitary conditions of his own making.

All the arguments that have been used for the existence of State health authorities are also available, it seems to me, for the creation and support of some organization for the protection of the public health in connection with the General Government. I think that the medical profession is agreed that no power less than that of the National Government will be sufficient to prevent the introduction of disease from abroad, or control its transport from State to State. There is, of course, a question as to what form that central authority should take and how extensive should be its power.

The well-being of the country, to use the language of the Constitution, should imply that we must know all that can be known about those diseases and causes of disease which may affect the whole country.

Although the actual epidemic prevalence of disease of grave and frequently fatal character may be limited to certain districts, still the disturbance of commerce and the ordinary relations of life will extend to large sections of the whole country. Therefore the National health authority should have the power and the means necessary for a thorough investigation of the communicable and epidemic diseases, and of the best means of preventing their spread. These investigations need not be limited to this country; in fact, it is quite evident that several of the most important diseases of this class must be also studied outside of the country, yellow fever, for instance.

Reports of the Committees on *Meteorological Conditions*, on *Fœticides* and on *Duties Commonly Exercised by Coroners*.

The Secretary then read the names of the newly-elected

OFFICERS OF SECTIONS.

*Practice of Medicine, etc.*—F. C. Shattuck, Boston, Massachusetts, Chairman; G. A. Fackler, Cincinnati, Ohio, Secretary.

*Surgery and Anatomy.* — N. P. Dandridge, Cincinnati, Ohio, Chairman; W. O. Roberts, Louisville, Kentucky, Secretary.

*Obstetrics and Diseases of Women.*—W. H. Wathen, Louisville, Kentucky, Chairman; A. B. Carpenter, Cleveland, Ohio, Secretary.

*State Medicine.*—J. Berrien Lindsley, Nashville, Tennessee, Chairman; S. T. Armstrong, U. S. Marine Hospital, New York, Secretary.

*Ophthalmology, Otology, and Laryngology.* —George E. Frothingham, Ann Arbor, Michigan, Chairman; G. C. Savage, Nashville, Tennessee, Secretary.

*Laryngology and Otology.*—W. H. Daly, Pittsburg, Pennsylvania, Chairman; E. Fletcher Ingals, Chicago, Illinois, Secretary.

*Diseases of Children.*—J. A. Larrabee, Louisville, Kentucky, Chairman; C. J. Jennings, Detroit, Michigan, Secretary.

*Medical Jurisprudence.*—J. G. Kiernan, Chicago, Illinois, Chairman; T. C. Evans, Baltimore, Maryland, Secretary.

*Dermatology and Syphilography.* — L. Duncan Bulkley, New York, Chairman; W. T. Corlett, Cleveland, Ohio, Secretary.

*Oral and Dental Surgery.*— F. H. Rehwinkel, Chillicothe, Ohio, Chairman; E. S. Talbot, Chicago, Illinois, Secretary.

The Secretary then stated that the Section on State Medicine recommends the passage of the following resolution by the Association.

*Resolved*, That the American Medical Association urges upon the House of Representatives of the Congress of the United States the necessity of and importance of the immediate passage of Senate Bill No. 2493—"*To Perfect the Quarantine Service of the United States*"— which bill has passed the Senate and is now pending in the House of Representatives, in order to make provision for protection against the introduction of contagious diseases before the approaching summer.

On motion, this resolution was adopted as the sense of the Association.

The following, for amending the bylaws, viz: "To change the by-law whereby the officers of sections are elected by the sections," was read, and lies over until the next meeting.

The amendment to the constitution offered by Dr. C. Seiler, Pennsylvania, in 1884, and now asked for by the Section on Ophthalmology, was called up and agreed to, and a new *Section on Laryngology and Otology* was created.

The Permanent Secretary read the list of members appointed as delegates abroad, as follows:

R. H. Plummer, San Francisco, California; N. S. Davis, Chicago, Illinois; J. J. Chisolm, Baltimore, Maryland; L. A. Sayre, New York, N. Y.; J. B. Hamilton, Washington, D. C.; V. C. Vaughan, Ann Arbor, Michigan; J. E. Owens, Chicago, Illinois ; S. J. Jones, Chicago, Illinois; H. A. Kelly, Philadelphia, Pennsylvania; A. E. Hoadley, Chicago, Illinois; W. H. Myers, Fort Wayne, Indiana; F. E. Waxham, Chicago, Illinois; J. V. Shoemaker, Philadelphia, Pennsylvania; D. A. K. Steele, Chicago, Illinois; Alex. McAlister, Camden, New Jersey; Ephraim Cutter, New York, N. Y.

After a formal vote of thanks to the presiding officers and to the profession and citizens of Cincinnati, who had done so much to make the meeting of the Association so agreeable, it adjourned.

